# The Impact of Clinical Pharmacist Implemented Protocol on Albumin Utilization and Cost in an Intensive Care Unit in Egypt

**DOI:** 10.3389/fphar.2022.825048

**Published:** 2022-03-18

**Authors:** Dina Mohamed Ibrahim, May Ahmed Shawki, Mohamed Hassan Solayman, Nagwa Ali Sabri

**Affiliations:** ^1^ Clinical Pharmacy Department, New Cairo Hospital, Cairo, Egypt; ^2^ Clinical Pharmacy Department, Faculty of Pharmacy, Ain Shams University, Cairo, Egypt

**Keywords:** human albumin, inappropriate use, drug use rationalization, cost reduction, pharmacist interventions

## Abstract

**Introduction:** Albumin is an expensive non-blood plasma substitutes with limited availability that has been reported to be inappropriately used in healthcare settings. Hence, interventions are recommended to control its misuse.

**Objective:** To evaluate the impact of clinical pharmacist implemented dispensing protocol on optimization of albumin use in an intensive care unit (ICU).

**Design:** A retrospective prospective 3-phase interventional study was conducted in an ICU in a tertiary Egyptian hospital over a period of 2 years.

**Methods:** The study included three phases; a preparation phase where a local albumin dispensing protocol and a restriction dispensing form were prepared by clinical pharmacists and was approved by the local Drugs and Therapeutics Committee, a retrospective pre-implementation phase in which the medical records of all ICU patients receiving albumin were evaluated for appropriateness of albumin use according to the developed protocol, and a prospective implementation phase where the dispensing protocol and restriction dispensing form were applied. The pattern of albumin consumption and cost were recorded and compared between the retrospective and prospective phases.

**Results:** In the retrospective phase, 190 ICU patients received albumin of whom 83.6% was considered inappropriate indications for albumin compared to only 44 patients in the prospective phase of whom 16% was considered inappropriate (*p*-value <0.001). Clinical pharmacists’ interventions significantly decreased the inappropriate albumin consumption from 4.7 vials/patient in the retrospective phase to 2.7 vials/patient in the prospective phase (*p*-value <0.001) with a total cost savings of 313,900 Egyptian Pounds (19,930 US Dollars).

**Conclusion:** The current study showed that clinical pharmacists’ interventions led to a significant control on albumin use and consequently reduced the cost associated with its consumption.

## Introduction

Drug misuse is increasingly recognized as a major challenge due to the associated unnecessary costs that burden the healthcare systems (Khamas et al., 2021). Several concerns are raised in medical practice, especially for drugs characterized by elevated cost and limited resources (Javan-Noughabi et al., 2020).

Human albumin is an expensive colloid derived from blood via a harsh production process. It has a superior advantage over non protein colloids for being a safe product, despite the probable risk of potential allergic reactions (Liumbruno et al., 2009; Zolfagharian et al., 2017; Dastan et al., 2018). However, albumin has been reported to be inappropriately used in several conditions. These include; management of malnutrition in critically ill patients, nephrotic syndrome not associated with hypovolemia and/or pulmonary edema, ascites responsive to diuretic therapy and pancreatitis (Liumbruno et al., 2009; Caraceni et al., 2013; Farasatinasab et al., 2018). Several studies have reported albumin misuse in the literature, showing its negative clinical and economic impacts (Lyu et al., 2016; Zolfagharian et al., 2017; Dastan et al., 2018; Ishida et al., 2018; Javan-Noughabi et al., 2020). This misuse could be attributed to absence of collective protocols defining appropriate albumin indications to guide healthcare providers, and also the lack of restrictive dispensing in the clinical setting (Buckley et al., 2020).

Human albumin utilization represents a critical area where clinical pharmacists can potentially improve clinical practice. Clinical pharmacists are drug-oriented healthcare practitioners who can play essential roles in optimizing medication use, improving patient outcomes and reducing additional costs from drugs misuse or unnecessary hospital stay that might result from inappropriate drug use (Chiatti et al., 2012; Noormandi et al., 2019). Few studies evaluated the role of clinical pharmacist interventions on albumin use in ICU and showed a positive clinical and economic impact (Dastan et al., 2018; Buckley et al., 2020).

This study aimed to evaluate the impact of clinical pharmacist implemented local protocol and restriction dispensing form on albumin use in an intensive care unit (ICU).

## Materials and Methods

### Methods

A retrospective prospective interventional study was conducted, at an ICU in a tertiary Egyptian hospital. The study included three phases: preparation phase, retrospective phase and prospective phase.

#### Preparation Phase

In this phase, the appropriate use criteria of human albumin were determined and a local dispensing protocol for albumin was prepared by clinical pharmacists based on available international evidence-based guidelines (Charles et al., 2008; Liumbruno et al., 2009; Weinacker and Ang, 2017). In addition, an albumin dispensing restriction form was prepared by the principal investigator. It comprised three parts; the first part included patient’s demographic data (age, weight, gender, serum albumin, and the required number of vials) in addition to physicians and pharmacists’ signatures. The second part comprised a list of inappropriate albumin indications and contraindications to its use, and the third part included a checklist form for appropriate albumin indications from which physicians could select the most appropriate indication depending on patient’s status. The protocol and the restriction form were approved by the hospital’s Drugs and Therapeutics Committee (DTC).

All healthcare team were educated about the approved indications of albumin and how to use the new dispensing form in several educational sessions. These sessions were held by clinical pharmacy department to ensure adherence to the approved protocol.

#### Retrospective Phase

From January 2018 till December 2018, medical records of all patients who was admitted to the ICU and prescribed albumin, were reviewed by the principal investigator. Data including demographic information, serum albumin level, indication of albumin prescription, the number of albumin vials dispensed, and the duration of albumin use were collected, and the cost of albumin used was calculated based on the Egyptian market price; 430 Egyptian Pounds per vial (27.3 US Dollars per vial). Indications for albumin use were categorized as either appropriate or inappropriate according to the developed dispensing protocol.

#### Prospective Phase

Starting from January 2019, no human albumin was dispensed from the hospital pharmacies unless the dispensing form was completely reviewed for appropriateness and the required signatures were fulfilled. Medical records of all patients who received albumin in this phase were reviewed by the principal investigator and the information was gathered in the same manner of the retrospective phase. Data extracted from this phase were compared to that from the retrospective phase.

### Statistical Analysis

Statistical analysis was done using IBM SPSS^®^ Statistics version 22 (IBM^®^ Corp., Armonk, NY, USA). Numerical data were expressed as mean and standard deviation (SD) or median and range as appropriate, while qualitative data were expressed as frequency and percentage. Pearson’s Chi-square test was used to examine the relation between qualitative variables. Comparison between the two phases was done using Student t-test for normally distributed quantitative data or Mann-Whitney test (non-parametric *t*-test) for not normally distributed quantitative data. The overall rate of consumption (vials/patient) and the cost of albumin vials (cost/patient) were compared assuming Poisson distribution using a Chi-square test-based method. All *p*-values were two sided and *p*-values <0.05 were considered significant.

### Ethics Approval

The study protocol was revised and approved by the research ethics committee for experimental and clinical studies of Faculty of Pharmacy, Ain Shams University-Cairo-Egypt (REC-ASU: approval No: 66/2018) which is approved and registered at Egyptian Ministry of Health (MOH), in addition to the approval of New Cairo Hospital managerial board on the study protocol and hospital’s DTC.

## Results

From January 2018 to December 2019, 234 patients were included in the study with a total of 998 human albumin vials being received during the study period. Eight hundred and sixty-two vials were dispensed to 190 out of 532 ICU patients (35.7%) in the retrospective phase versus 136 vials dispensed for 44 out of 679 ICU patients (6.4%) in the prospective phase.

### Baseline Demographic and Clinical Data

The age of the patients who received albumin in the study ranged from 16 to 90 years. Fifty three percent (n = 101) of patients in the retrospective phase were males compared to 50% (n = 22) in the prospective phase. The most common cause for ICU admission was diabetic ketoacidosis and respiratory distress syndrome in the retrospective and prospective phases respectively. Baseline serum albumin was significantly higher in the retrospective phase compared to the prospective phase (*p*-value = 0.002). Demographic and clinical data of ICU patients who were included in the study in the two phases are represented in Table 1.

**TABLE 1:** Baseline characteristics of the retrospective and prospective phases.

Parameter	Retrospective phase n = 190	Prospective phase n = 44	*p*-value
Age (years): Mean ± SD	56.7 ± 18.9	58.8 ± 20	0.512^a^
Gender			0.705^b^
Male: Number (%)	101 (53.2%)	22 (50%)	
Female: Number (%)	89 (46.8%)	22 (50%)	
Cause of admission: Number (%)			
Chronic Kidney disease	8 (4.2%)	0 (0%)	#
Cirrhotic Ascites	13 (6.8%)	4 (9.1%)	
Diabetic Ketoacidosis	39 (20.5%)	7 (15.9%)	
Malnutrition	25 (13.2%)	1 (2.3%)	
Nephrotic Syndrome	21 (11.1%)	6 (13.6%)	
Pancreatitis	5 (2.6%)	0 (0%)	
ARDS	23 (12.1%)	18 (40.9%)	
Sepsis	9 (4.7%)	4 (9.1%)	
Shock	20 (10.5%)	4 (9.1%)	
Stroke	27 (14.2%)	0 (0%)	
Baseline serum albumin; Mean ± SD	2.5 ± 0.6	2.2 ± 0.4	0.002^a^ ^*^

a ARDS: Acute respiratory distress syndrome a: Statistical test; Student t- test.

b Statistical test; Pearson’s Chi square test.

#*p*-value cannot be calculated.

*indicates significance.

### Albumin Utilization Data

#### Inappropriate Albumin Indications

In the retrospective phase, 159 patients out of 190 received albumin for inappropriate indications representing 83.7%. Using Pearson’s Chi square test, this was significantly decreased in the prospective phase where only seven patients out of 44 (15.9%) received albumin inappropriately (*p*-value < 0.001). In the retrospective phase, the most common inappropriate albumin indications were hypoalbuminemia and malnutrition. Detailed inappropriate human albumin indications in the retrospective and the prospective phases is represented in Table 2.

**TABLE 2:** Inappropriate human albumin indications in the retrospective and the prospective phases.

Indications	Retrospective phase		Prospective phase	
	Number of patients	Inappropriate indications n (%)	Number of patients	Inappropriate indications n (%)
ARDS	5	0 (0%)	8	0 (0%)
Ascites responsive to diuretics	6	6 (100%)	—	—
Cirrhotic Ascites	7	6 (85.7%)	6	0 (0%)
Hemodialysis	2	2 (100%)	—	—
Hemorrhagic Shock	3	0 (0%)	3	0 (0%)
HRS Type1	2	0 (0%)	4	0 (0%)
Hypoalbuminemia	79	72 (91.1%)	1	0 (0%)
Malnutrition	46	46 (100%)	1	1 (100%)
Nephrotic Syndrome	14	11 (78.6%)	10	6 (60%)
No Serum Albumin check	7	7 (100%)	—	—
Pancreatitis	5	5 (100%)	—	—
SBP	5	0 (0%)	7	0 (0%)
Septic Shock	5	0 (0%)	4	0 (0%)
Volume resuscitation in hypovolemia	4	4 (100%)	—	—
Total number of patients (%)	190	159 (83.7%)	44	7 (15.9%)

ARDS: Acute respiratory distress syndrome, HRS: Hepatorenal syndrome, SBP: Spontaneous bacterial peritonitis

#### Number of Inappropriate Albumin Vials Dispensed

The median (range) of dispensed vials of albumin per patient was 4 (1–9) and 2.5 (1–6) in the retrospective and prospective phases respectively. Comparison between the two phases with respect to the median number of vials used per patient using Mann Whitney test, revealed significant difference (*p*-value < 0.001). In the retrospective phase, 862 vials were dispensed, of which 749 vials were dispensed of inappropriate indications (86.9%). In the prospective phase, only 136 vials of albumin were dispensed of which only 19 were considered inappropriate (14%), with an absolute reduction of inappropriate albumin use of 72.9% between the retrospective and the prospective phases.

Using Poisson distribution method, the inappropriate albumin vials consumption significantly decreased from 4.7 vials/patient in the retrospective phase to 2.7 vials/patient in the prospective phase (*p*-value <0.001). The detailed number of albumin vials dispensed for each indication in both the retrospective and the prospective phases is shown in Table 3.

**TABLE 3:** Total and inappropriate albumin vials dispensed in the retrospective and prospective phases.

Indications	Retrospective phase		Prospective phase	
	Total number of albumin vials dispensed	Inappropriate use	Total number of albumin vials dispensed	Inappropriate use
		Number of vials (%)		Number of vials (%)
ARDS	13	0 (0%)	27	0 (0%)
Ascites responsive to diuretics	36	36 (100%)	—	—
Cirrhotic Ascites	39	35 (89.7%)	17	0 (0%)
Hemodialysis	4	4 (100%)	—	—
Hemorrhagic Shock	5	0 (0%)	4	0 (0%)
HRS Type1	7	0 (0%)	14	0 (0%)
Hypoalbuminemia	425	393 (92.5%)	5	0 (0%)
Malnutrition	184	184 (100%)	2	2 (100%)
Nephrotic Syndrome	68	57 (83.8%)	29	17 (58.6%)
No Serum Albumin check	10	10 (100%)	—	—
Pancreatitis	22	22 (100%)	—	—
SBP	22	0 (0%)	29	0 (0%)
Septic Shock	19	0 (0%)	9	0 (0%)
Volume resuscitation in hypovolemia	8	8 (100%)	—	—
Total number of vials	862	749 (86.9%)	136	19 (14%)

ARDS: Acute respiratory distress syndrome, HRS: Hepatorenal syndrome, SBP: Spontaneous bacterial peritonitis

#### Duration of Albumin Use per Patient

Comparison of the duration of albumin use was done by Mann Whitney test. The mean (±S.D.) duration of albumin use per patient significantly decreased from 2.4 (±0.9) days in the retrospective phase to 1.8 (±0.8) days in the prospective phase (*p* -value <0.001).

#### Cost Evaluation of Inappropriate Albumin Vials Dispensed

The cost of inappropriate albumin vials dispensed was estimated to be 322,070 Egyptian Pounds (20,449 US Dollars) and 8,170 Egyptian Pounds (519 US Dollars) in the retrospective and the prospective phases respectively. The total wasted cost due to inappropriate albumin use saved due to the clinical pharmacists’ interventions was estimated to be 313,900 Egyptian Pounds (19,930 US Dollars). Using Poisson distribution method, the cost of inappropriate albumin vials dispensed/patient was significantly reduced from 2025.6 Egyptian Pounds/patient (128.6 US Dollars/patient) in the retrospective phase to 1,167 Egyptian Pounds/patient (74.1 US Dollars/patient) in the prospective phase, (*p*-value <0.001).

## Discussion

The current study focused on albumin use in ICU setting because it is a unique setting where different specialty healthcare providers work together and are subjected to physically and emotionally challenging environments that is usually overwhelming (Ervin et al., 2019). Critically ill patients are also diverse in nature of their diseases, and they frequently suffer from complex comorbidities and multiple organs disorders. As a consequence, polypharmacy is usually present, and drug misuse is commonly recorded in practice in ICU (Nibrad et al., 2015; Ervin et al., 2019; Religioni and Pakulska, 2020).

Few studies highlighted the impact of clinical pharmacist on optimizing albumin use in ICU but they only depended on the development of albumin protocol by clinical pharmacists (Farsad et al., 2016; Lyu et al., 2016). In addition to the development of approved protocol, Buckley et al., mentioned that clinical pharmacists assessed all albumin orders for appropriateness and interfered when necessary (Buckley et al., 2020).

The current study was built on three major pillars. The first one was the development of “Albumin use protocol” similar to previous studies. The clinical pharmacists collected the evidence-based criteria for albumin use based on the latest guidelines and designed an institution specific protocol for albumin use and dispensing which presented a comprehensive guide for physicians to help in the decision-making process of appropriate albumin prescription. In addition, two other interventions were suggested in the current study that has not been evaluated previously. One of them is the use of restriction form designed by the principal investigator who is the head of clinical pharmacy department to control albumin dispensing. As a member of the DTC, the principal investigator discussed the albumin use protocol and its restriction form in the DTC meeting and obtained the approval of the DTC and the hospital board manger. A decision was issued to the hospital pharmacists not to dispense albumin vials to the ICU unless the restriction form was fulfilled and signed by both the prescribing physician and one of the clinical pharmacy team. A meeting with the hospital pharmacists was held to discuss with them the new restriction form. Hospital pharmacists had the responsibility of revising the dispensing orders and had the authority to refuse dispensing albumin if the restriction sheet was not attached to prescription or if the required signatures were not obtained.

The third and the most critical pillar was physicians’ education to ensure long term sustainable results. Physicians are the cornerstone of the healthcare team (Ervin et al., 2019), thus providing proper education is essential to improve the quality of patient care (Foroughinia and Mazraie, 2017). The educational sessions conducted by clinical pharmacy team aimed to enhance awareness of the physicians about the importance of the implemented protocol and the expected potential benefits on healthcare cost reduction to enhance their compliance to the approved protocol. The current work adopted active learning techniques to promote higher decision making, through interactive, team-based discussions with the physicians and all healthcare team. The Clinical pharmacy team, who was responsible for educating healthcare providers and following up the application of the protocol, included 15 clinical pharmacists, with years of experience ranged from 5 to 14 years. All clinical pharmacists hold postgraduate degrees including Clinical Pharmacy diploma (n = 11), master’s degree in Clinical Pharmacy (n = 2), and master’s and philosophy doctor degrees in Clinical Pharmacy (n = 1). All clinical pharmacy team receives regular annual training conducted by the Egyptian MOH.

In the retrospective phase of the current work, the misuse of albumin represented 83.7% of the indications according to the implemented protocol. Several other Iranian studies demonstrated similar albumin misuse percentages in the ICU, ranging from 36.2% to 95% (Shafiee et al., 2011; Talasaz et al., 2012; Kazemi et al., 2013; Farsad et al., 2016; Farasatinasab et al., 2018). This was also the same reported in the study of Buckley et al., in the United States where the inappropriate albumin use in the ICU were 63.4% at baseline (Buckley et al., 2020). This confirms that albumin misuse is an international problem.

The current study has shown that the strategies implemented by the clinical pharmacists significantly increased the appropriateness of albumin use in the ICU from 16.3% in the retrospective phase to 84.1% in the implementation phase. In addition, inappropriate albumin consumption has significantly decreased by 72.9%. Buckley et al., and Lyu et al., similarly reported an absolute decrease in albumin misuse by 50.9% and 36% respectively following the use of approved protocols (Lyu et al., 2016; Buckley et al., 2020). The great results in the current work compared to the previous studies might be attributed to the combined interventions used by the clinical pharmacists emphasizing the role of education and setting rules that control physician prescribing. At baseline, physicians showed high resistance to changing the prescribing pattern of albumin. They perceived filling and signing of the restriction forms as a burden, regarding it as an unnecessary time-consuming step. After educating them about the importance of rationalizing albumin prescribing, the potential benefits that would be reflected on the patients’ outcomes and the general cost savings that would be obtained, physicians were very cooperative during the prospective phase, and they supported the implementation of the program. This emphasizes the importance of healthcare professionals’ education.

One of the most commonly listed reasons for irrational albumin use is hypoalbuminemia with serum albumin level above 2.5 g/dl not associated with hypovolemia (Caraceni et al., 2013; Farasatinasab et al., 2018; Javan-Noughabi et al., 2020). Similar finding was detected in the current work, where hypoalbuminemia represented 45.3% of the total inappropriate albumin indications in the retrospective phase. Moreover, the current study showed that baseline serum albumin was significantly higher in the retrospective phase compared to the prospective one, indicating the inappropriate use of albumin in the retrospective phase. This could be explained by the fact that physicians did not pay enough attention to the patients’ albumin serum level. Similar results were mentioned in previous studies evaluating albumin use appropriateness in ICUs, where hypoalbuminemia represented from 23.4% to 36.2% of the inappropriate indications (Shafiee et al., 2011; Talasaz et al., 2012; Farsad et al., 2016; Farasatinasab et al., 2018).

It was revealed that the second most common cause of inappropriate albumin use in the current study was malnutrition, which represented 28.9% of the total inappropriate albumin indications. Similarly, previous studies in Iran (Shafiee et al., 2011; Farasatinasab et al., 2018) reported that almost 100% of the albumin use as supplemental protein in the ICU was irrational. Despite the worldwide available evidence that discourages using albumin as a protein supplement in nutritional support of critically ill patients, albumin is still being used widely in nutritional regimens (Caraceni et al., 2013; Zolfagharian et al., 2017). This may be attributed to the complicated procedures of ordering and preparation of total parenteral nutrition formulas (Singer et al., 2019). Consequently, physicians order albumin to avoid the tedious time consuming processes (Zolfagharian et al., 2017). Administration of amino acids and sufficient caloric diet formulas through enteral or parenteral nutrition can achieve better outcomes in critically ill patients who suffer nutritional problems (Mehta et al., 2018; Singer et al., 2019). In addition, rapid and sharp elevation of serum albumin levels to above 4 g/dl may lead to increase in the overall rate of catabolism in the body and may results in failure in therapeutic process (Liumbruno et al., 2009; Shafiee et al., 2011; Farasatinasab et al., 2018).

Drug misuse imposes huge financial burden on the governments and healthcare sector, and on the patients themselves (Chiatti et al., 2012; Soleymani et al., 2015; Ofori-Asenso and Agyeman, 2016). Buckley et al., mentioned that the relative reduction in the cost of total albumin use decreased by 34.8%, and that of inappropriate albumin use decreased by 87.1% in the ICU after the use of approved protocols (Buckley et al., 2020). In the current study, the combined strategies used led to around 84.2% relative cost reduction in the total albumin use and 97.5% in the inappropriate albumin use, emphasizing the impact of combined strategies implemented by clinical pharmacist in controlling the misuse associated cost in the ICU.

## Conclusion

The current study showed that the use of clinical pharmacist-led multiple strategies had a great impact on albumin consumption rates and significantly decreased the waste in money dispensed in albumin misuse in the ICU.

## Data Availability

The data that support the findings of this study are available from the corresponding author upon reasonable request.

## References

[B1] Buckley M. S. Knutson K. D. Agarwal S. K. Lansburg J. M. Wicks L. M. Saggar R. C. (2020). Clinical Pharmacist-Led Impact on Inappropriate Albumin Use and Costs in the Critically Ill. Ann. Pharmacother. 54, 105–112. 10.1177/1060028019877471 31544470

[B2] Caraceni P. Domenicali M. Tovoli A. Napoli L. Ricci C. S. Tufoni M. (2013). Clinical Indications for the Albumin Use: Still a Controversial Issue. Eur. J. Intern. Med. 24 (8), 721–728. 10.1016/j.ejim.2013.05.015 23790570

[B3] Charles A. Purtill M. Dickinson S. Kraft M. Pleva M. Meldrum C. (2008). Albumin Use Guidelines and Outcome in a Surgical Intensive Care Unit. Arch. Surg. 143 (10), 935–939. 10.1001/archsurg.143.10.935 18936370

[B4] Chiatti C. Bustacchini S. Furneri G. Mantovani L. Cristiani M. Misuraca C. (2012). The Economic burden of Inappropriate Drug Prescribing, Lack of Adherence and Compliance, Adverse Drug Events in Older People: a Systematic Review. Drug Saf. 35 (1), 73–87. 10.1007/BF03319105 23446788

[B5] Dastan F. Jamaati H. Emami H. Haghgoo R. Eskandari R. Hashemifard S. S. (2018). Reducing Inappropriate Utilization of Albumin: The Value of Pharmacist-Led Intervention Model. Iran J. Pharm. Res. 17 (3), 1125–1129. 30127835 PMC6094441

[B6] Ervin J. N. Kahn J. M. Cohen T. R. Weingart L. R. (2019). Teamwork in the Intensive Care Unit. Am. Psychol. 73 (4), 468–477. 10.1037/amp0000247 PMC6662208 29792461

[B7] Farasatinasab M. Amouzegar A. Safari S. Ghanbari B. Darkahian M. Emami S. (2018). Albumin Utilization Evaluation in a Major Teaching Hospital in Iran: Recommendations for Guideline Development. J. Res. Pharm. Pract. 7 (3), 157–163. 10.4103/jrpp.JRPP_18_4 30211241 PMC6121762

[B8] Farsad B. Hadavand N. Masumi S. Salehi H. (2016). Albumin Utilization Review to Evaluate the Efficacy and Cost, Perform as a Qualitative Study in Special Wards in Shaheed Rajaei Cardiovascular, Medical &Research Center. Biosci.,Biotech.Res. 13 (3), 1469–1477. 10.13005/bbra/2290

[B9] Foroughinia F. Mazraie S. (2017). Investigating the Use of Human Albumin in a Non-teaching Hospital in Iran. Iran J. Pharm. Res. 16 (2), 817–822. 28979337 PMC5603893

[B10] Ishida T. S. Sakai M. C. de Melo D. O. (2018). The Appropriate Use of Human Albumin in a Brazilian University Hospital: Therapeutic Indication and Dosage Regimen. Braz. J Pharm Sci [Internet] 54 (4), 10. 10.1590/s2175-97902018000418008

[B11] Javan-Noughabi J. Parnian E. Hajiesmaeili M. Salehiniya H. Setoodehzadeh F. (2020). The Impact of a Guideline to Prevent Inappropriate Albumin Administration in a Hospital in Iran. Br. J. Heal Care Manag. [Internet] 29, 26. 10.12968/bjhc.2019.0086

[B12] Kazemi Y. Hadavand N. Hayatshahi A. Torkamandi H. Gholami K. Hadjibabaie M. (2013). Albumin Utilization in a Teaching Hospital in Tehran: Time to Revise the Prescribing Strategies. J. Pharm. Care 1 (4), 127–132.

[B13] Khamas S. S. Mirbagheri I. Dehnadi-moghaddam A. Ashouri A. (2021). Evaluation of Albumin Utilization in a Major Teaching Hospital in Iran before and after Guideline Implementation. J. Pharm. Care 9 (2), 67–73. 10.18502/jpc.v9i2.6609

[B14] Liumbruno G. M. Bennardello F. Lattanzio A. Piccoli P. Rossettias G. (2009). Recommendations for the Use of Albumin and Immunoglobulins. Blood Transfus. 7 (3), 216–234. 10.2450/2009.0094-09 19657486 PMC2719274

[B15] Lyu P. F. Hockenberry J. M. Gaydos L. M. Howard D. H. Buchman T. G. Murphy D. J. (2016). Impact of a Sequential Intervention on Albumin Utilization in Critical Care. Crit. Care Med. 44 (7), 1307–1313. 10.1097/CCM.0000000000001638 26963324

[B16] Mehta Y. Sunavala J. D. Zirpe K. Tyagi N. Garg S. Sinha S. (2018). Practice Guidelines for Nutrition in Critically Ill Patients: A Relook for Indian Scenario. Indian J. Crit. Care Med. 22, 263–273. 10.4103/ijccm.IJCCM_3_18 29743765 PMC5930530

[B17] Nibrad V. V. Nayak B. Raul A. Vijayprasad S. Vakade K. Jadhav A. (2015). Drug Utilization Pattern in Medical Intensive Care Unit ( MICU ) in a Tertiary Care Teaching Hospital in Rural Area of Maharashtra. Int. J. Appl. Biol. Pharm. Technol. [Internet] 4, 148–153. 10.18203/2319-2003.ijbcp20151347

[B18] Noormandi A. Karimzadeh I. Mirjalili M. Khalili H. (2019). Clinical and Economic Impacts of Clinical Pharmacists' Interventions in Iran: a Systematic Review. Daru 27, 361–378. 10.1007/s40199-019-00245-8 30674033 PMC6593130

[B19] Ofori-Asenso R. Agyeman A. A. (2016). Irrational Use of Medicines-A Summary of Key Concepts. Pharmacy (Basel) 4 (4), 35. 10.3390/pharmacy4040035 PMC5419375 28970408

[B20] Religioni U. Pakulska T. (2020). Rational Drug Use in Hospital Settings - Areas that Can Be Changed. J. Med. Econ. 23 (10), 1205–1208. 10.1080/13696998.2020.1801455 32715825

[B21] Shafiee E. Rezaee H. Entezari T. Hamishehkar H. (2011). The Evaluation of Albumin Use in an Iranian University Hospital. Pharm. Sci. 22 (3), 186–189. 10.15171/ps.2016.29

[B22] Singer P. Blaser A. R. Berger M. M. Alhazzani W. Calder P. C. Casaer M. P. (2019). ESPEN Guideline on Clinical Nutrition in the Intensive Care Unit. Clin. Nutr. 38 (1), 48–79. 10.1016/j.clnu.2018.08.037 30348463

[B23] Soleymani F. Haerizadeh M. Farshchi A. (2015). Economic burden of Irrational Use of Injectable Form of Dexamethasone: a Warning to Health System. J. Pharmacoeconomics Pharm. Manag. [Internet] 1 (2), 56–58. Available from: http://jppm.tums.ac.ir/index.php/jppm/article/view/58.

[B24] Talasaz A. H. Jahangard-Rafsanjani Z. Ziaie S. Fahimi F. (2012). Evaluation of the Pattern of Human Albumin Utilization at a university Affiliated Hospital. Arch. Iran Med. 15 (2), 85–87. 22292577

[B25] Weinacker A. Ang H. (2017). ALBUMIN Indications. Stanford Heal Care [Internet], 1–4. Available from: https://stanfordhealthcare.org/content/dam/SHC/health-care-professionals/medical-staff/medstaff-weekly/20170315-guidelines-for-intravenous-albumin-administration.pdf.

[B26] Zolfagharian F. Ghazanfari S. Elyasi S. Iraji P. Saberi M. R. Vahdati-Mashhadian N. (2017). Drug Utilization Evaluation of Albumin in a Teaching Hospital of Mashhad, Iran: an Interventional Pre-post Design Study. Int. J. Clin. Pharm. 39 (4), 704–711. 10.1007/s11096-017-0458-y 28540466

